# Special Issue: Mucosal Fungal Infections

**DOI:** 10.3390/jof4020043

**Published:** 2018-03-26

**Authors:** Jonathan P. Richardson, Julian R. Naglik

**Affiliations:** Mucosal & Salivary Biology Division, Dental Institute, King’s College London, London WC2R 2LS, UK; jonathan.richardson@kcl.ac.uk

**Keywords:** mucosal infection, fungus, *Aspergillus*, *Candida*, *Cryptococcus*, Mucorales, mycobiome

The past four decades have seen a staggering escalation in the number of invasive fungal infections worldwide. Pathogenic fungi pose an ever-increasing threat to human health, and the global impact of mucosal-acquired fungal infections on healthcare regimens is of major concern. Collectively, fungal infections have an unacceptably high mortality rate of ~40%. The yearly healthcare expenditure for the treatment of fungal diseases is estimated to be $15 billion, thus the global economic and societal impact that pathogenic fungi exert can no longer be ignored. The ongoing battle against fungal pathogens in the clinical setting is hampered by poor and/or time-consuming diagnostics, a limited repertoire of anti-fungal drugs, increasing levels of resistance to anti-fungal therapies, and (somewhat alarmingly) the absence of a vaccine against any known fungal pathogen [1]. 

Species of *Candida*, *Aspergillus*, and *Cryptococcus* are among several common fungal pathobionts that cause life-threatening infections in millions of immunocompromised individuals worldwide each year. Collectively, these species (and many others) constitute a massive global disease burden that will continue to increase in prevalence and severity with the passing of time should current and future strategies of intervention fail. Indeed, the incidence of invasive fungal infections is already rising, and the number of deaths caused each year by these infections is equal to or greater than the number of deaths caused by malaria or tuberculosis [2].

The majority of systemic fungal infections are acquired across the mucosal surfaces, thus it is of paramount importance to better understand the mechanisms by which fungi interact with epithelial tissues and how the immune system seeks to restrict these fungi to mucosal surfaces during health. This Special Issue of *Journal of Fungi* highlights the recent advances being made in the field of fungal-epithelial interactions and immunity against mucosal-acquired fungal infections. Eight review articles [3,4,5,6,7,8,9,10] comprise this Special Issue, which highlight the impact of *Aspergillus*, *Candida*, *Cryptococcus*, Mucorales and the mycobiome on human health.

The mucosal surfaces of the body are colonised by a diverse microbiota. Tremendous advances have been made in our understanding of the microbes that colonise mucosal surfaces and while the predominant number of colonising microbial species are bacterial in origin, the role of fungi in these polymicrobial communities is significant. The changes in fungal population dynamics within the microbiota, and the role of these changes in disease are explored by Witherden et al. [3]. The authors discuss techniques used to study the mycobiome and compare the mycobiomes of oral and gastrointestinal environments. Polymicrobial interactions between components of the mycobiota and the surrounding microbiota are considered together with strategies used to model these processes and interactions with the host, using the latest cutting-edge computational approaches.

Approximately 20 species of *Candida* fungi are known to be pathogenic to humans. Localised overgrowth of *Candida* fungi on mucosal surfaces results in the development of candidiasis at disparate mucosal sites in the body (oral, gastrointestinal, vaginal), causing morbidity in otherwise healthy individuals, and deep-seated invasive infections that threaten mortality in the absence of effective immune defence. The direct and indirect mechanisms used by *Candida* species to colonise, damage, and breach mucosal barriers are discussed by Richardson et al. [4] together with a review of secreted virulence factors and their contribution to mucosal interactions. This includes recent findings relevant to the activity of candidalysin, the first fungal peptide toxin identified in any human fungal pathogen. The host immune response to invasive fungi is a critical determinant of clearance from the body. Recent years have seen significant advances in our understanding of innate mucosal immunity to *C. albicans*. In their review article, Verma et al. [5] explore the innate mechanisms of epithelial defence against *Candida* fungi with an emphasis on responses to *C. albicans*. The authors evaluate the role of soluble host factors (cytokines, chemokines, antimicrobial peptides, and alarmins) in effective defence and highlight the role of innate immunity mediated by haematopoietic cells.

*Aspergillus fumigatus* is a common airborne saprophytic fungus. During its life cycle, *A. fumigatus* releases conidia into the atmosphere that are subsequently inhaled into the lungs where they cause a complex range of debilitating respiratory infections in patients with compromised immunity. In their review, Bertuzzi et al. [6] explore the temporal and mechanistic events that underpin attachment and uptake of *A. fumigatus* by the respiratory epithelium, the contribution of these processes to respiratory aspergilloses, and epithelial responses to the fungus. Differential cellular responses to *A. fumigatus* morphotypes are discussed by the authors as well as current opinions regarding the role of epithelial uptake as a curative or pathogenic process. The adaptive T-Helper (Th) immune responses to *A. fumigatus* are explored in detail by Dewi et al. [7], who review the factors that influence effective adaptive clearance (Th1/Th17) and allergic hypersensitivity/detrimental immune pathology (Th2/Th17). The authors also discuss the role of regulatory T-cells in this context and explore the challenges associated with the development of effective immunotherapy.

The basidiomycete *Cryptococcus neoformans* causes disease in otherwise healthy individuals and those with compromised immunity. As the onset of cryptococcosis is typically associated with HIV positive individuals and hence defective adaptive immunity, mucosal defence against *C. neoformans* is somewhat understudied by comparison. Taylor-Smith [8] reviews the interactions that occur between *C. neoformans* and epithelial cells that lead to fungal adherence and internalisation into the respiratory mucosa. The importance of bronchial and alveolar epithelial responses in driving innate defence against infecting fungus is explored together with a discussion of fungal responses in the respiratory niche. The recent advances in adaptive immunity to *C. neoformans* are considered by Mukaremera and Nielsen [9], who discuss the complex and varied cellular and antibody responses to the fungus. The authors also evaluate the role of protective cytokines, and present current models that describe adaptive immune responses to *C. neoformans* in mice and humans.

Fungi of the order Mucorales (predominantly *Rhizopus*, *Mucor*, and *Lichtheimia* species) are the cause of mucormycosis, an aggressive, devastating invasive fungal disease with a mortality rate of 50–100%. The innate and adaptive immune defence mechanisms that are brought to bear against Mucorales are discussed by Ghuman and Voelz [10], who describe the role of immune cells and platelets in antifungal defence together with the challenges faced when clearing Mucorales from the body, a process whose efficacy is influenced by the developmental stage of Mucorales sporangiospores.

While understudied compared with bacterial infections, the devastating impact of invasive fungal infections on the global population can no longer be ignored. Given the massive increase in the use of therapeutic interventions that result in the suppression of host immunity (for example, chemotherapy and solid organ transplantation) combined with an ageing population, a greater understanding of fungi-host interactions together with more efficacious therapeutics and diagnostics are needed now more than ever. It is our collective hope that continued research will improve our understanding and manipulation of fungal pathogenicity and host defence mechanisms that may lead to the discovery of new approaches to prevent these fungal infections, thus significantly enhancing the therapeutic armoury.

We would like to take this opportunity to thank all of the authors that contributed to this Special Issue of *Journal of Fungi*. Your time and expertise is appreciated.
